# ADHD and Substance Misuse in the Club Drug Clinic: A Case Series

**DOI:** 10.1192/bjo.2025.10768

**Published:** 2025-06-20

**Authors:** Abiram Selladurai, Urbah Viqar, Owen Bowden-Jones

**Affiliations:** CNWL, London, United Kingdom

## Abstract

**Aims:** Attention Deficit Hyperactivity Disorder (ADHD) has a well-established link with substance misuse, with growing evidence that individuals with ADHD are at higher risk of developing addictive behaviours including substance use disorders.

The Club Drug Clinic is a specialised addiction service providing support for individuals experiencing problems related to the use of club drugs. During assessments and reviews, patients presenting with symptoms suggestive of ADHD are formally assessed. Those who are diagnosed with ADHD are then commenced on appropriate treatment.

**Methods:** 15 out of 98 patients (15%) under the service since July 2023 were diagnosed with ADHD by the Club Drug Clinic, all of whom were subsequently commenced on atomoxetine.

Data was collected from patient notes including: age, gender, sexuality and information about their substance misuse. The doses of atomoxetine prescribed as well as side-effects and perceived benefits was also recorded. Information around co-morbid mental illness was also analysed.

**Results:** 87% of patients in this series were male with the majority of patients reporting their sexuality as either homosexual or bisexual which is reflective of the population group served generally by the Club Drug Clinic. The age range of the patients was 24–58 with an average age of 40.

Crystal Methamphetamine was the most frequently used illicit drug with 70% of those diagnosed with ADHD using Crystal Meth in an either a harmful or dependent manner. 60% of those with ADHD were using GHB often in conjunction with Crystal Meth. 50% of those identified in this study were using ketamine.

47% of patients commenced on atomoxetine reported side-effects and 33% of patients commenced on atomoxetine stopped due to side-effects or a lack of perceived benefit.

Depression was the most common co-morbidity in those diagnosed with ADHD seen in 20% of this cohort. Other co-morbidities include: anxiety disorder, borderline personality disorder, bipolar affective disorder and interestingly gaming disorder.

**Conclusion:** This series reflects the strong association between ADHD and addiction particularly within the Club Drug Clinic population. Implications include routine screening for ADHD in Club Drug users to help identify cases early and potentially reduce long-term substance misuse. It also highlights the importance of integrated mental health services given the high rates of co-morbidity in patients suffering from addiction.

